# Supplementary material for: Validity and Reliability of Kinect v2 for Quantifying Upper Body Kinematics during Seated Reaching
Source: Sensors (Basel). 2022 Apr 2;22(7):2735. doi: 10.3390/s22072735 (PMC9003545; doi:10.3390/s22072735)
Supplement: Supplementary file 1 [file sensors-22-02735-s001.zip › sensors-1622458-supplementary.pdf]

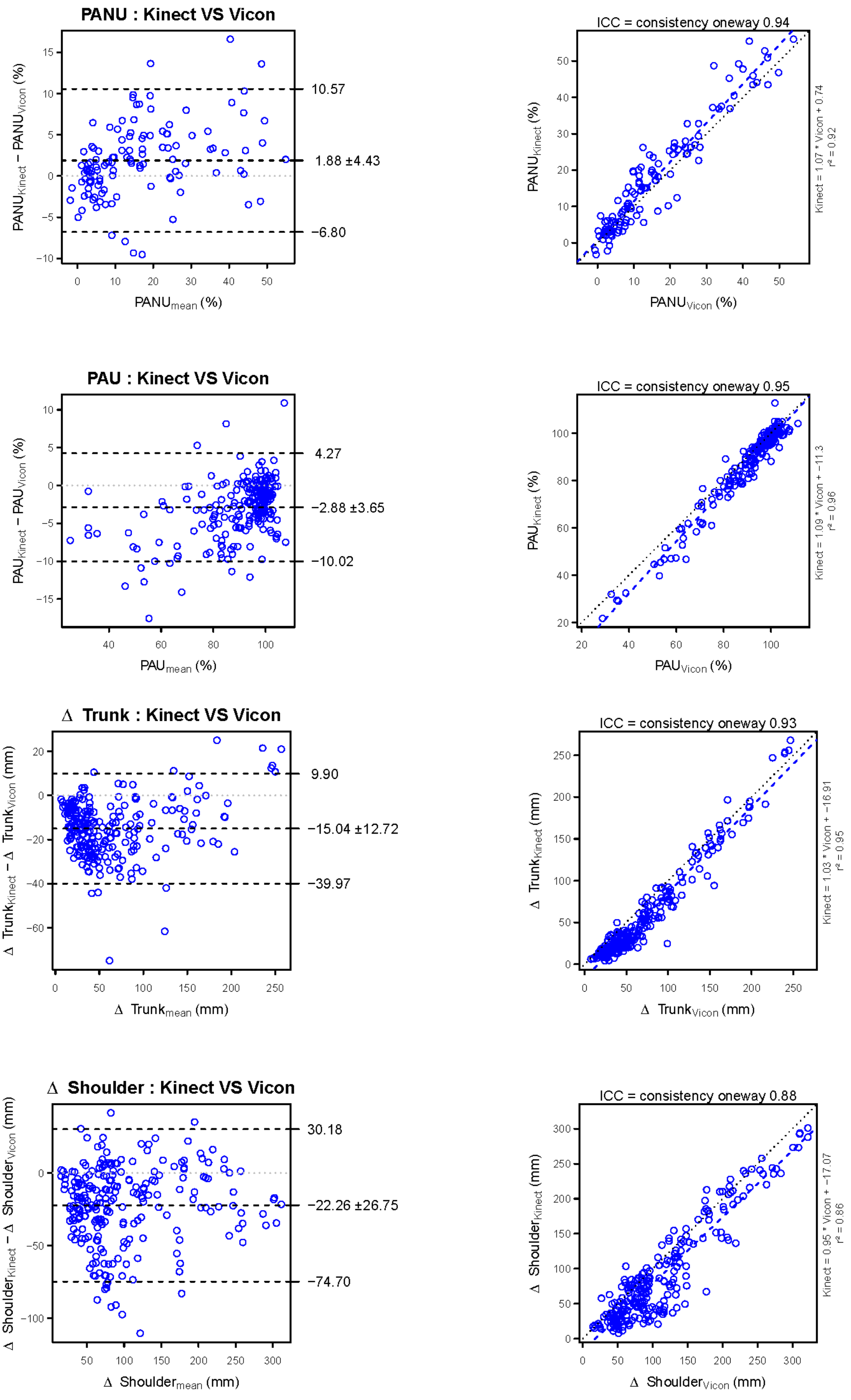
PANU : Kinect VS Vicon
PANU_Kinect − PANU_Vicon (%)
PANU_mean (%)
10.57
1.88 ±4.43
−6.80
ICC = consistency oneway 0.94
PANU_Kinect (%)
PANU_Vicon (%)
Kinect = 1.07 * Vicon + 0.74
r² = 0.92
PAU : Kinect VS Vicon
PAU_Kinect − PAU_Vicon (%)
PAU_mean (%)
4.27
−2.88 ±3.65
−10.02
ICC = consistency oneway 0.95
PAU_Kinect (%)
PAU_Vicon (%)
Kinect = 1.09 * Vicon + −11.3
r² = 0.96
Δ Trunk : Kinect VS Vicon
Δ Trunk_Kinect − Δ Trunk_Vicon (mm)
Δ Trunk_mean (mm)
9.90
−15.04 ±12.72
−39.97
ICC = consistency oneway 0.93
Δ Trunk_Kinect (mm)
Δ Trunk_Vicon (mm)
Kinect = 1.03 * Vicon + −16.91
r² = 0.95
Δ Shoulder : Kinect VS Vicon
Δ Shoulder_Kinect − Δ Shoulder_Vicon (mm)
Δ Shoulder_mean (mm)
30.18
−22.26 ±26.75
−74.70
ICC = consistency oneway 0.88
Δ Shoulder_Kinect (mm)
Δ Shoulder_Vicon (mm)
Kinect = 0.95 * Vicon + −17.07
r² = 0.86

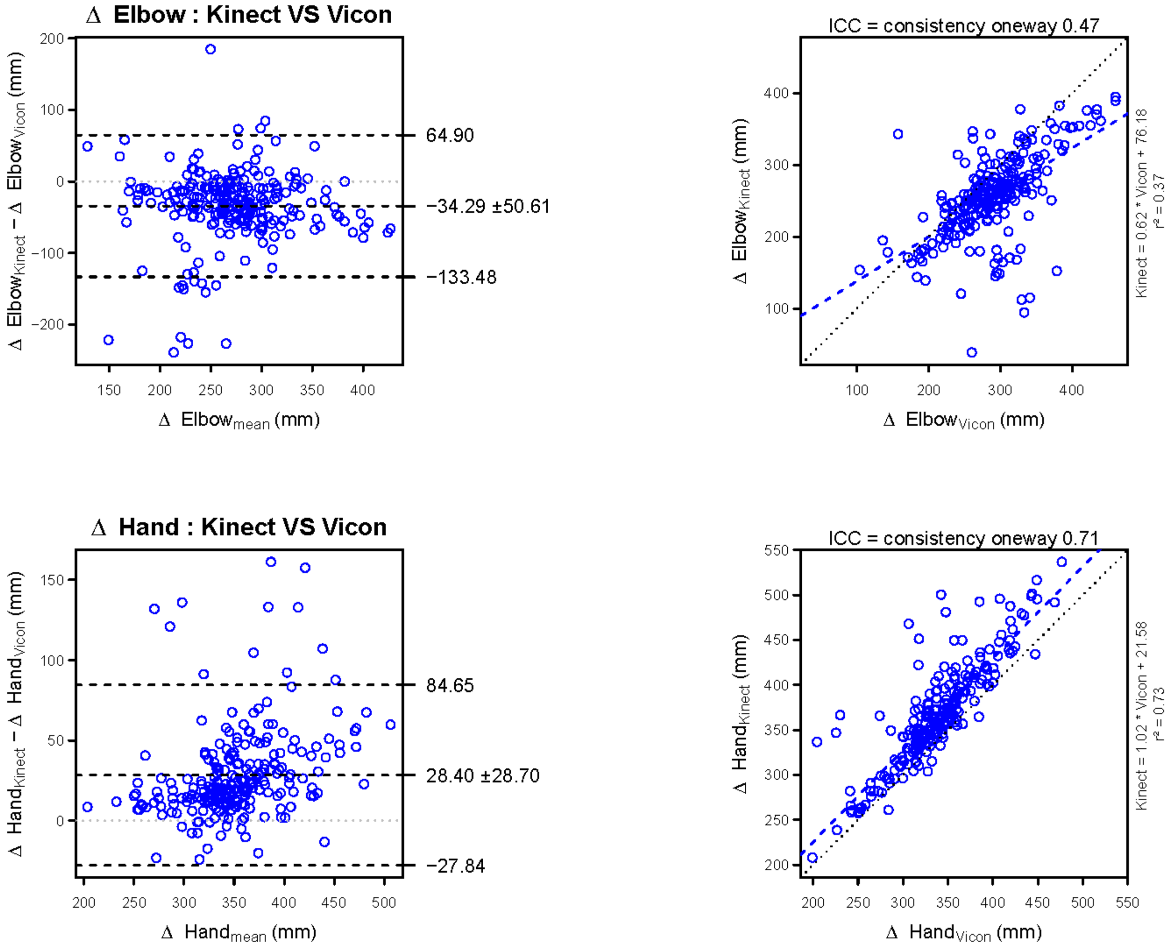

Figure S1. Comparison of nonuse and joint displacements assessed with the Kinect and the Vicon systems. Panels in the first row illustrate PANU. Panels in the second row illustrate proximal arm use (PAU. Panels in the third row illustrate trunk displacement (Δ Trunk). Panels in the fourth row illustrate shoulder displacement (Δ shoulder). Panels in the fifth row illustrate elbow displacement (Δ elbow). Panels in the sixth row illustrate hand displacement (Δ hand). For each row, the left panel represents the Bland and Altman plot, and the right panel represents the linear regression plot. When assessed with the Kinect, PANU is excellently reliable but slightly overestimated, PAU is excellently reliable and very slightly underestimated, Δ trunk is excellently reliable and slightly underestimated, Δ shoulder is goodly reliable and slightly underestimated, Δ elbow is poorly reliable and slightly underestimated, Δ hand is moderately reliable and very slightly overestimated.
